# Invasion of Chicken Intestinal Cells Is Higher for *Enterococcus cecorum* Lesion Strains Compared to Cloacal Strains in an Organoid Model

**DOI:** 10.3390/microorganisms13010050

**Published:** 2024-12-31

**Authors:** Lonneke Vervelde, Thijs T. M. Manders, Samira Kammourieh, Jeanine Wiegel

**Affiliations:** 1 Department Poultry Health, Royal GD, 7418 EZ Deventer, The Netherlands; t.manders@gddiergezondheid.nl (T.T.M.M.); s.kammourieh@gddiergezondheid.nl (S.K.); j.wiegel@gddiergezondheid.nl (J.W.); 2 Department of Population Health Sciences, Faculty of Veterinary Medicine, Utrecht University, 3584 CL Utrecht, The Netherlands

**Keywords:** *Enterococcus cecorum*, organoids, virulence, translocation, intestine

## Abstract

Some strains of *Enterococcus cecorum* can cause spondylitis and bacterial osteomyelitis. Translocation and bacteremia are pivotal to the pathogenesis and clinical disease. Virulence typing to distinguish extra-intestinal disease of lesion from cloacal strains remains difficult. We investigated if organoids can be applied to differentiate between *E. cecorum* strains that are more or less virulent. Floating chicken intestinal organoids combine the complex cell system of the gut with an easily accessible apical-out orientation. The organoids were treated with four *E. cecorum* strains that differ in original isolation, lesion, or cloacal, and bacterial load was determined after 3 and 6 h by quantitative PCR and bacterial plating. Independent of the inoculum dose or time post inoculation, DNA levels of *E. cecorum* marginally differed between the strains. To determine if this was caused by adherence of bacteria to the epithelial cells, an invasion assay was developed. The organoids were inoculated with the different *E. cecorum* strains and after 3 or 6 h treated with an antimicrobial mixture, lysed, and quantified by bacterial plate counting. Significantly higher (*p* < 0.0001) numbers of bacteria isolated from lesions invaded the organoids compared to cloacal strains in a dose-dependent manner. Higher numbers of bacteria isolated from lesions invaded the organoids compared to cloacal strains in a dose-dependent manner. This study is a major step in the development of a model to study the interaction between *E. cecorum* and the chicken host and a model to test novel intervention strategies to prevent translocation of bacteria.

## 1. Introduction

*Enterococcus cecorum* are members of the normal enterococcal microbiota in the gastrointestinal tract of poultry and are gram-positive, facultatively anaerobic, non-spore forming cocci. *E. cecorum* infections have emerged since the early 2000s as a significant challenge in poultry production worldwide, and clinical outbreaks can have major economic impact through septicemia, spondylitis, and bacterial osteomyelitis, which subsequently lead to locomotor disorders and increased mortality rates of up to 15%, while condemnation rates up to 9.8% have been reported [1,2,3].

Strains are usually considered virulent when they are recovered from typical *E. cecorum* lesions in femur and free thoracic vertebra, whereas commensal or avirulent strains are generally isolated from the intestines and cloaca of healthy birds. To assess the virulence of an *E. cecorum* strain, ideally all strains should be typed in the target animal, as it is important to determine the ability to reproduce clinical signs and typical gross lesions caused by the *E. cecorum*-isolate in an animal challenge model. These models are informative; however, typical lesions are only induced in a low-to-moderate percentages of birds, making in vivo typing of *E. cecorum* expensive due to the large number of birds required to obtain relevant results [4,5,6,7,8]. In addition to in vivo typing, various genotypic and phenotypic methods have been applied to distinguish lesion from cloacal *E. cecorum* strains. Compared to cloacal strains, the lesion strains caused higher cumulative embryo mortality in chicken lethality embryo assays [9,10,11,12]. Moreover, lesion strains have been shown to be more resistant to high concentrations of lysozyme [13], but none of the aforementioned in vitro phenotypic methods are conclusive. Genetic diversity between pathogenic or non-pathogenic has been shown using pulsed-field gel electrophoresis, molecular typing of virulence genes, and genome-wide comparisons. Nonetheless, studies that clearly distinguish distinct lesion strains from cloacal strains based on genetic information are lacking [1,14,15,16,17,18,19].

The pathogenesis of *E. cecorum* is not well understood and knowledge gaps that have to be addressed further include how and where the bacteria translocate, how do bacteria circumvent the immune system to set up persistent infections, and what is the natural host-pathogen relationship [5,7]. In vivo models for *E. cecorum* are being improved, but the variable and low-to-moderate incidence of systemic infection rates hampers in depth in vivo investigation of the pathogenesis [4,5,8,20]. Alternative in vitro models that aim to recapitulate the intestinal environment vary in cellular complexity; the simplest cell lines and primary intestinal epithelial cultures mimic select features of the intestine but lack cellular, anatomical and physiological complexity. The 2D and 3D organoids represent the cellular complexity of the intestinal epithelium, but the most complex cellular models are the gut explants and the floating chicken intestinal organoid model (reviewed in Nash and Vervelde 2022) [21]. The floating chicken intestinal organoid model was demonstrated to contain representative gut epithelial populations of intestinal stem cells, Paneth cells, enteroendocrine cells, goblet cells, and enterocytes and contained the original lamina propria cells because they are grown from isolated villi instead of single stem cells and represent the 3D architecture of the intestine [22]. Moreover, they can be infected with bacteria (*Salmonella*), viruses (avian influenza virus), and the protozoan parasite *Eimeria tenella* [22]. These characteristics demonstrated a superior representation of the intestinal physiology compared to 2D intestinal organoids in a *Salmonella* challenge model, because the presence of immune cells in 3D organoids regulated the inflammatory responses induced by *Salmonella*, while this regulatory effect of the lamina propria cells was lacking in 2D organoids [23].

In addition, Mitchell et al. (2024) [24] demonstrated that the chicken intestinal organoids are a novel tool to measure the effect of feed additives in a bacterial challenge model. In this study, we evaluated if the organoid model can be used to investigate potential differences in translocation of cloacal and lesion *E. cecorum* strains. We hypothesized that by using in an invasion assay and four well characterized *E. cecorum* strains [12], we could distinguish between lesion strains that have the potential to invade the organoids and cloacal strains that would not invade organoids. This model will enable us to address outstanding questions on pathogenesis, translocation, and local immune responses to *E. cecorum*, and in addition facilitate screening of novel preventive and therapeutic intervention strategies in vitro before in vivo trials are performed.

## 2. Material and Methods

### 2.1. Generations of Chicken Intestinal 3D Organoids

Experiments were performed using 18-day-old embryos of specific pathogen free White Leghorns (*Gallus gallus*) bred at Royal GD, Deventer, the Netherlands. Embryos were humanely culled under the guidance of the Central Authority for Scientific Procedures on Animals, according to the Dutch Animal Procedure Act.

The generation of 3D intestinal organoids has been described previously [22]. In brief, the small intestine and caeca were removed, cut open longitudinally, then into 3 mm sections and collected in Mg^2+^ and Ca^2+^ free phosphate buffer saline (PBS; Gibco, Paisley, UK). For each batch, the intestines from three to five embryos were pooled to form independent batches or biological replicates. The villi were released from the tissue with *Clostridium histolyticum* type IA collagenase (0.2 mg/mL, Sigma, Cream Ridge, NJ, USA) at 37 °C and purified using a 70 μM cell strainer (Greiner, Kremsmünster, Germany). The villi were collected by washing the inverted strainer and pelleted at 100 g for 4 min. The pellet was resuspended in Floating Organoid Media (FOM) consisting of Advanced Dulbecco’s Modified Eagle Medium (DMEM)/F12 (Gibco, Paisley, UK) supplemented with B27 (ThermoFisher Scientific, Paisley, UK), 10 mM HEPES (Gibco, Paisley, UK), 2 mM L-Glutamine and 50 U/mL Penicillin/Streptomycin (Gibco, Paisley, UK), and incubated overnight in petri dishes at 37 °C, 5% CO_2_. The non-adherent organoids were removed, washed in DMEM, and resuspended in FOM without Penicillin/Streptomycin. The organoids were seeded at ~1100 organoids/well in 6-well-plates and further cultured for 1 or 2 days at 37 °C, 5% CO_2_ [25] until inoculation with *E. cecorum*.

### 2.2. Selection of E. cecorum Strains

The *E. cecorum* strains used in this study are available at Royal GD and were previously characterized by Manders et al. (2022, 2024) [12,13] and summarized in Table 1. The four *E. cecorum* strains were selected based on the site of isolation, ability to ferment mannitol, embryo mortality rate in an embryo lethality assay, and the resistance against lysozyme (Table 1). In brief, two *E. cecorum* strains were isolated from cloacal swabs of healthy broiler reproduction animals (cloacal stains), and two strains were isolated from broilers with spondylitis and femur head necrosis (lesion strains). All strains have been sequenced and were not clonally related.

### 2.3. Antimicrobial Susceptibility Testing of the Strains

Antimicrobial susceptibility of *E. cecorum* isolates was tested with broth microdilution method for bacteria isolated from animals described for enterococci in the Clinical and Laboratory Standard Institute (CLSI) with the addition of lysed horse blood and under microaerophilic conditions [26]. Minimal inhibitory concentrations (MICs) of penicillin, streptomycin, and gentamicin were determined in serial two-fold dilutions using commercially manufactured, customized MIC plates (Merlin Diagnostics, Bornheim-Hersel, Germany). MIC values were determined by reading the plates with a photometer (Merlin Diagnostics) and interpreting the results with the advanced expert system (AES) MCN-6 software of Merlin (Merlin Diagnostics; version 6.00 27-11-2023 rel. 126).

### 2.4. Inocula Preparation

*E. cecorum* strains were recovered from cryopreservation (15% glycerol stocks at −80 °C), plated on Columbia agar supplemented with sheep blood (SBA, BioTrading, Mijdrecht, The Netherlands), and incubated at 37 °C in a 5% CO_2_-enriched atmosphere. After 24 h of incubation, one colony per strain was scraped off, replated on SBA, and incubated overnight for maximum 48 h. To prepare the inoculum, several colonies were scraped of the plate and suspended in physiological salt solution to an optical density of 0.5 McFarland (McFarland Densitometer, type DEN-1, Grant instruments Ltd., Royston, UK), which corresponds to approximately 10^8^ colony-forming units (CFU)/mL. The bacterial concentrations of the inocula were assessed by means of bacterial colony counting of ten-fold serial dilutions on SBA.

### 2.5. Bacterial Infection of Organoids

The organoids cultures were first inoculated with 10^7^ CFU/well *E. cecorum*, and the experiment was repeated twice with 3 biological replicates. At 3 and 6 h post inoculation organoids were harvested for further analysis. Based on the outcome, the experiments were repeated with 10^3^, 10^5^ and 10^6^ CFU/well with 3 biological repeats.

To determine bacterial load by quantitative RT-PCR, organoids were harvested, washed thrice in PBS, and resuspended in 230 μL PBS. DNA was isolated using MagMAX Pathogen RNA/DNA kit (Applied Biosystems, Paisley, UK) according to manufacturer’s instructions. *E. cecorum* DNA quantification was based on superoxide dismutase (*SodA*) gene using the AgPath-ID One-step RT-PCR kit (Applied Biosystems) with forward primer GATGAAGAAACAATGCATCTACAT, reverse primer GGCGTCATAATTTTCCAGAAGAATG, and probe ATAACAGCTTGGCGAATATC. Amplification and detection of specific products were carried out with an Applied Biosystems Quantstudio 5 Realtime cycler (Waltham, MA, USA) with the following cycle profile, 10 min 45 °C, 10 min 95 °C, and 45 cycles of 95 °C for 15 s and 60 °C for 45 s.

An invasion assay was developed to determine translocation of bacteria. After inoculation with *E. cecorum* and incubation for 3 or 6 h as described above, the organoids were treated with a mixture of penicillin/streptomycin (1000 U/mL, TFS, Paisley, UK) and gentamicin (50 µg/mL, Dechra, Bladel, The Netherlands) for 30 min at 37 °C, 5% CO_2_. The organoids were harvested, washed thrice in PBS, and resuspended in 500 μL 0.5% Triton-X100 in PBS (Merck Millipore, Darmstadt, Germany) to lyse the organoids and incubated for 30 min at room temperature. The lysates were serially diluted 10^−1^ to 10^−3^ and plated on SBA, incubated at 37 °C and 5% CO_2_ and counted the next day. The number of live bacteria that invaded the organoids was calculated and expressed as CFU/mL. A similar approached was followed as we employed for the RT-qPCR analysis, starting with 10^7^ CFU/well, and the invasions experiments were repeated thrice with three biological replicates in each experiment; and for the lower inocula, three biological replicates were used. A replicate was regarded as contaminated by external factors and discarded if another bacterium was detected on the SBA plate which is a non-selective growth medium.

### 2.6. Statistical Analysis

For the Ct values and bacterial concentrations (CFU/mL), data are presented as mean values with standard deviation or 95% confidence intervals. The Shapiro–Wilk test was used to check for normality of the data. Students *T*-test was used to compare the 45-Ct values obtained in the organoids inoculated with 10^7^ and 10^5^ CFU, the Man–Whitney test for the other two groups. The bacterial invasion data of lesion strains compared to cloacal strains were analyzed by Mann–Whitney test. The probability level for significance was taken as *p*  <  0.05. Statistical analysis of the data was performed by using GraphPad Prism version 10.2.3 for windows [27].

## 3. Results

### 3.1. Effect of E. cecorum on Intestinal Organoid Morphology

**Figure 1:**
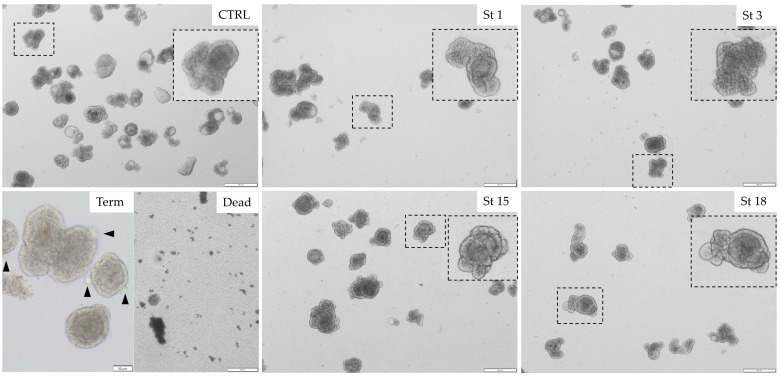
Representative images of organoids grown for 3 days in floating organoid medium (FOM). Organoids are inoculated with 10^7^ CFU per well with strain (St) 1, 3, 15, 18 or FOM (CTRL) and imaged at 6 h post inoculation. Cloacal strains 1 and 3 are described as avirulent and lesion strains 15 and 18 as virulent strains (Manders et al., 2022) [12]. Enlargements of healthy organoids are given in dashed rectangles to demonstrate the morphology after treatment. For comparison, organoids grown for 48 h in DMEM only (Term) start to dissociate and show excess shedding of enterocytes (arrowheads), while organoids grown for 72 h in DMEM only (Dead) fully disintegrate. Scale bars: 200 μm and Term 50 μm.

To investigate the effect of *E. cecorum* on the morphology of chicken intestinal organoids, we inoculated the cultures with 10^7^ CFU per well and imaged the organoids 6 hpi. Independent of the bacterial strain, the morphology of the organoids was not affected by a high inoculum dose of *E. cecorum* (Figure 1). Substantial budding of the organoids indicated rapid growth which was seen independent of the treatment. Lower inocula doses and the 3 h time point showed similar growth characteristics.

### 3.2. Quantification of E. cecorum by Quantitative PCR in Chicken Intestinal Organoids

**Figure 2:**
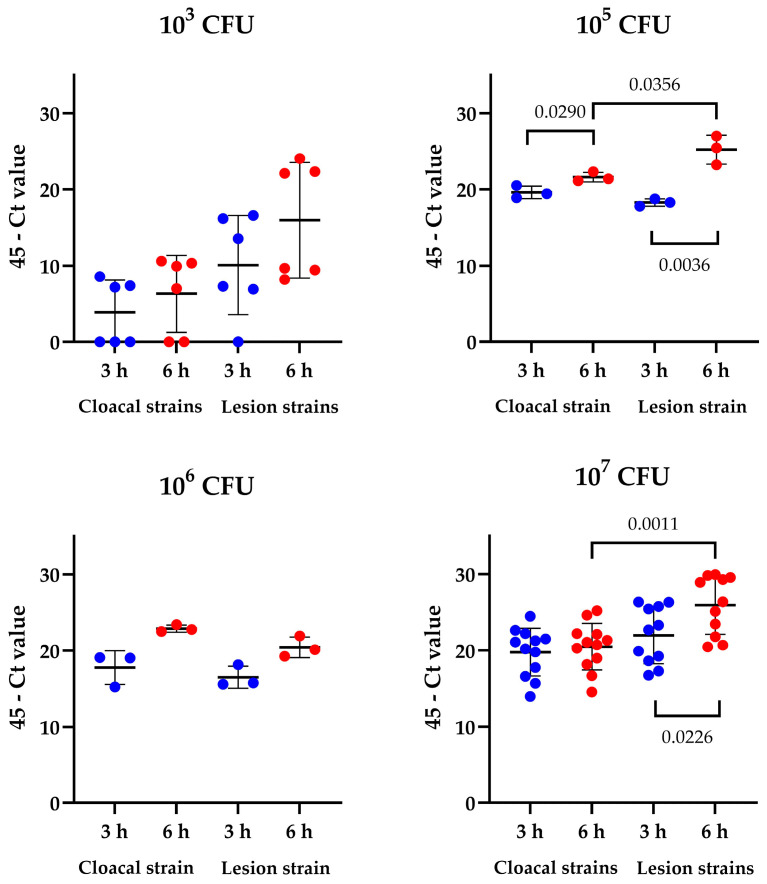
Quantification of *E. cecorum* by quantitative PCR in chicken intestinal organoids inoculated with 10^3^ to 10^7^ CFU per well. The organoids were harvested at 3 (red) or 6 (blue) hours post inoculation. Significant differences between cloacal and lesion strains were analyzed by Student’s *T*-test. *N* = 3 for 10^5^ to 10^6^ CFU/well and *N* = 6 for 10^3^ and N = 11–12 for 10^7^ CFU/well. Data are presented as means with standard deviation.

To investigate if *E. cecorum* lesion strains translocated at a higher rate than the cloacal strains, we first measured bacterial load by quantitative PCR. The organoids were inoculated with 10^3^ to 10^7^ CFU per well, and bacterial load was quantified at 3 and 6 h post inoculation (Figure 2). There were no differences between cloacal and lesion strains after inoculation of 10^3^ CFU. This low inoculation dose resulted in inconsistent invasion of all strains with not all replicates *E. cecorum* positive (Table 2).

Significant differences between cloacal and lesion strains were detected after inoculation with 10^5^ but not with 10^6^ CFU, although the bacterial DNA levels at 6 h were consistently higher compared to 3 h after inoculation. At the highest dose of 10^7^ CFU, a significant difference was detected between cloacal and lesions strains and, for the lesion strains, there was also a significant effect of time. Strain specific data are given in Table 2.

### 3.3. Quantification of E. cecorum by Invasion Assay in Chicken Intestinal Organoids

**Figure 3:**
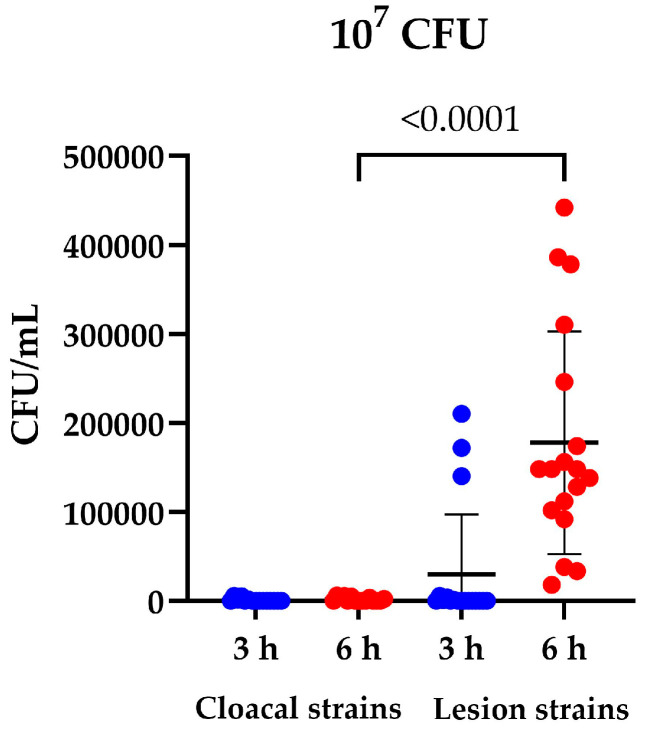
Invasion assay using chicken intestinal organoids inoculated with 10^7^ CFU *E. cecorum* per well. The organoids were harvested at 3 (red) or 6 (blue) hours post inoculation, treated with an antimicrobial mixture, lysed, and quantified by bacterial plate counting. Significant differences between cloacal and lesion strains were analyzed by Mann–Whitney test (*p*  < 0.0001). Data are presented as means with standard deviation. *N* = 15 cloacal strains and *N* = 18 for lesion strains.

Despite multiple washing steps, we cannot exclude bacteria adhering to the organoids. Since quantification by qPCR cannot distinguish between adherent bacteria and invaded bacteria, we then developed an invasion assay to quantify invaded or translocated *E. cecorum*. The organoids were inoculated with 10^7^ CFU per well, and after 3 and 6 h the organoids were treated with an antimicrobial mixture, lysed and bacteria were quantified by plate counting. The number of bacteria recovered after antimicrobial treatment of the organoids differed substantially between the cloacal and lesion strains (Figure 3). At 6 h post inoculation, the lesion strains invaded the organoids to a significantly higher (*p*  < 0.0001) extend compared to the cloacal strains 1 and 3. At 3 h post inoculation, the variation within a group was too large to distinguish between the strains (Table 3).

**Figure 4:**
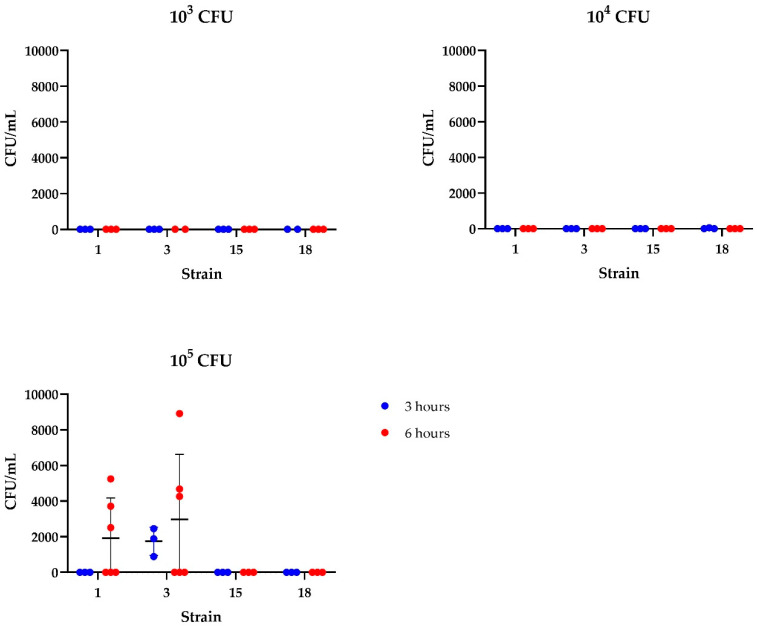
Invasion assay using chicken intestinal organoids inoculated with 10^3^ to 10^5^ CFU *E. cecorum* per well. The organoids were harvested at 3 (red) or 6 (blue) hours post inoculation, treated with an antimicrobial mixture, lysed, and quantified by bacterial plating. Cloacal strains 1 and 3 are described as avirulent and lesion strains 15 and 18 as virulent strains (Manders et al., 2022) [12]. *N* = 2–6. Data are presented as means with standard deviation.

Chaguza et al. (2023) [28] previously described that translocation of *E. faecalis* is dependent on overgrowth in the intestinal tract. Therefore, we tested the effect of lower concentrations of *E. cecorum* on invasion of the intestinal organoids. The number of bacteria recovered after antimicrobial treatment indicated a profound dose-dependent effect on translocation of different *E. cecorum* strains with a low reproducibility using 10^5^ CFU (Figure 4).

## 4. Discussion

The colonization of the intestine followed by translocation and bacteremia is of utmost importance to the pathogenesis of *E. cecorum* and development of lesions. The ability of *E. cecorum* to translocate from the intestinal tract to various tissues seems linked to virulence. This process of translocation has not been studied in detail due to a lack of a suitable in vitro chicken model. In this study, we investigated if chicken intestinal organoids can be applied to investigate translocation and to differentiate between cloacal and lesion *E. cecorum* strains based on invasion of the organoids. Our study revealed that based on an invasion assay and subsequent bacterial plating, lesion strains that have been characterized as more virulent based on embryo lethality assays, mannitol fermentation and resistance to lysozyme, invaded the organoids in contrast to cloacal strains. Using quantitative PCR we could not discriminate between the cloacal and lesion strains, which might be due to the ability of *E. cecorum* to adhere to the epithelial cells independent of their virulence or lack thereof. Also, quantitative PCR detects all bacterial DNA in the sample, bacteria adhered to epithelial cells, intracellular, and also live and dead bacteria. Bacterial plating only detects bacteria that were alive. Combined with the antibiotic treatment, this assay is a better representation of numbers of bacteria that invaded the organoid than quantitative PCR.

Previously, translocation of enterococci was investigated using mammalian cell lines. Zeng et al. [29] used a two-chamber system to study transcytosis of *E. faecalis* isolates across T84 cells, a human colon carcinoma cell line, and demonstrated marked differences between strains in their ability to translocate. Moreover, disruptive mutations in the enterococcal polysaccharide antigen (*epa*) gene cluster, known to be needed for virulence and resistance to killing by polymorphonuclear leukocytes, negatively affected the ability to translocate.

Disrupted cell junctions or damage to the intestinal barrier have also been suggested to facilitate translocation of *E. cecorum*. Schreier et al. [20] reported a trend toward more reisolations of *E. cecorum* in heat-stressed chickens compared to non-heat-stressed challenged chickens and decreased mRNA expression of tight junction genes claudin-5 (*CLDN5*) and tricellulin or marvelD2 (*MD2*) at 7 days post inoculation. Borst et al. [6] reported that coinfection with intestinal pathogens, i.e., coccidia, increased gut inflammation and reduced bacteremia and spinal lesions. However, no effect on the proportion of typical lesions was found by Manders et al. [30] after inoculating *E. cecorum* in combination with several avian pathogens, although the number of broilers with positive reisolations was higher when *E. cecorum* was inoculated in combination with chicken anemia virus or *Mycoplasma synoviae* in combination with infectious bronchitis virus or Newcastle disease virus. The foregoing suggests that reduced barrier integrity is not the sole cause for translocation. In our study, *E. cecorum* translocated over an apparently intact epithelial layer of the organoids based on microscopical examination of the organoids. Functional integrity of the visually intact epithelial layer should be assessed by for instance permeability assays.

Other *Enterococcus* spp., especially *E. faecalis*, are known for their ability to adhere to mammalian epithelial cells and the bacteria are taken up by a wide range of cells (reviewed in Archambaud et al., 2024) [31]. Several studies have looked for virulence traits and genes specifically associated with lesion strains of *E. cecorum*. Some candidate genes were identified that could discriminate between lesion and cloacal strains. Different genes encoding for metabolic enzymes were found in *E. cecorum* lesions strains. These alternative metabolic capacities may give these strains an advantage in competing over commensal strains in the gastrointestinal tract [14,15]. Genes encoding for capsular polysaccharides, which are involved in evasion of phagocytosis, have mainly been found in *E. cecorum* lesion strains. Furthermore, the enterococcal polysaccharide antigen (*epa*) locus was found to be highly conserved in lesion strains [14]. This antigen is thought to be involved in resistance to killing by phagocytosis and tissue invasion as described by Zeng et al. [29] to be of importance for *E. faecalis*. Enterococci can be internalized by more than one pathway, among which are receptor-mediated endocytosis or micropinocytosis [32,33]. How *E. cecorum* adheres and enters epithelial cells of chickens and through what mechanism is unknown. Further research will reveal what cellular processes are induced when *E. cecorum* adheres and invades chicken organoids.

After finding a difference between the cloacal and lesions strains used in this study, we investigated if the bacterial load affected the level of translocation. Archambaud et al. [34] demonstrated that, in a mouse model of bacterial dysbiosis, the distribution of the intestinal translocation data did not increase linearly with the level of enterococci in the intestinal lumen, but rather occurred once a threshold value had been reached. In our study, we also showed a clear dose-dependent effect before significant invasion is reached. This high dose-dependent threshold is not linked to the characteristics of the floating 3D chicken organoids because they can be infected by *Salmonella enterica* serovar Typhimurium strain 4/74 at a lower CFU/organoid and the invasion by STm 7/74 is a much faster process with ample invasion within 3 h after inoculation [24]. Bacterial loads of *E. cecorum* in vivo are unknown. However, based on the levels of enterococci measured by Archambaud et al. [34] in the intestinal content of mice and the quantitative PCR results of different locations of the intestines of broilers of Jung et al. [35], it is likely that the threshold can be reached in vivo as well. Further research is needed to confirm this.

*E. cecorum* colonizes the intestines of broilers from the first week of life in flocks experiencing an *E. cecorum* outbreak in later in the production cycle [5,35]. This pattern of early colonization supports the use of organoids derived from 18-day-old embryos that are matured for 3–4 days rather than the use of organoids from older chickens. When human preterm intestinal organoid monolayers were exposed to an *E. faecalis* with pathogenic potential, a distinct gene expression profile compared with adult organoids was found, but the level of invasion was not [36].

To ensure that only bacteria that invaded the organoids were cultured, a mixture of three different antimicrobials was used prior to bacterial examination. The antimicrobial mixture should be adjusted to the susceptibility of the isolates and preferable, bactericidal antimicrobials with a fast working mechanism should be used. These antimicrobials should not affect the organoids (physically and functionally) after exposure with high concentrations. Antimicrobial susceptibility profiles of *E. cecorum* are diverse and regional differences are seen [37,38]. There are no official breakpoints for *E. cecorum* published by EUCAST or CLSI [26]. Applicability of breakpoints for other *Enterococcus* spp. to *E. cecorum* is uncertain [39]. If no clinical or pharmacological breakpoints are available, epidemiological cut-off values (ECOFFs) can be used to determine whether susceptibility is decreased compared to the wild-type population. Unfortunately, officially recognized ECOFF values for *E. cecorum* are not available [39]. Therefore, it was not possible to select antimicrobials based on accepted susceptibility cut offs. Based on published distributions of antimicrobial susceptibility of *E. cecorum* isolates [1,16,37,38,40] and suggested tentative ECOFFs [40], it could be estimated that the four isolates did not have lower susceptibilities compared to published results and results of *E. cecorum* isolates from the Netherlands, for at least one of the three antimicrobial drugs used in the assay. All strains could be considered as susceptible to penicillin; however, the mechanism of action of penicillin is largely time dependent and bactericidal action starts after a lag period and, therefore, growth characteristics of the different strains can be of influence on the efficacy of antimicrobial inhibition in this assay. The activity of streptomycin and gentamicin is increased by coadministration with β-lactams; therefore, the activity of these two drugs may also be influenced by growth characteristics of the strains [41]. In future experiments, the antimicrobial mixture could be further optimized to the assay. However, choices clearly hampered by the lack of cut-off values for translation of MIC to susceptible or resistant.

Current methods for prevention and treatment of *E. cecorum* are limited. The standard treatment is administration of antimicrobials. However, treatment with antimicrobials will be ineffective for osteomyelitis already caused and only can be effective in septicaemic birds. Multidrug resistance is relatively common and matches the global rise [40,42] and, therefore, multiple alternative strategies are explored. Examples of these strategies are vaccinations of breeder flocks with mono- or polyvalent killed vaccines, but these vaccines do not protect their progeny after experimental challenge [7]. Other alternative strategies can include the use of bacteriophages, probiotics, and phytobiotics. Bacteriophages can kill bacteria in a highly selective manner without disturbing the normal microbiota in the intestinal tract. Limited studies using phages have been performed on *E. cecorum* and in vivo studies are lacking. The species *E. faecium* is used as a probiotic supplement in poultry diets [43] with beneficial effect when given preventively but not therapeutically [44]. Phytobiotics such as essential oils have good antimicrobial properties due to bacteriostatic and antibacterial substances, e.g., lactones, esters, and terpenes, and are effective against *Enterococcus* spp. in vitro [45]. The effectiveness of those prophylactic supplements needs to be tested against several virulent *E. cecorum* isolates. The intestinal organoid model, as described, might be used as a screening model for the effectiveness of those supplements or bacteriophages.

In future studies, this novel *E. cecorum* chicken model can be used to address knowledge gaps on *E. cecorum* host-pathogen interaction, translocation process, mucosal immune responses, and it can be applied to test novel intervention strategies.

## 5. Conclusions

In conclusion, we developed an in vitro model that differentiates between cloacal and lesion strains of *E. cecorum*. The model combines the use of floating chicken intestinal 3D organoids and an invasion assay, and we demonstrated a dose and time dependent invasion of virulent strains isolated from lesions.

## Figures and Tables

**Table 1:** Summary of the origin and characteristics of the *Enterococcus cecorum* inoculation strains used.

	Origin					MIC (mg/L)		
Isolate	Site	Lesion	Mannitol Fermentation	Embryo Mortality ^2^	Lysozyme MIC ^3^ Concentration (µg/mL) ^4^	Penicillin	Streptomycin	Gentamycin
1	Cloaca	- ^1^	+	0%	4000	0.0625	8	4
3	Cloaca	-	+	0%	8000	0.125	>16	≤1
15	FTV ^5^	Spondylitis	-	100%	Resistant ^6^	0.125	8	8
18	Femur	Femur head necrosis	-	89–100%	Resistant	0.0625	16	8

^1^ No lesions observed. ^2^ Range of cumulative embryo mortality is given, three embryo lethality assays were performed and described by Manders et al. (2022) [12]. ^3^ Minimum inhibitory concentration. ^4^ MIC concentrations determined by Manders et al. (2024) [13]. ^5^ Free thoracic vertebra. ^6^ Resistant to the maximum tested concentration of 8000 µg/mL.

**Table 2:** Results of *Enterococcus cecorum* PCR tests (45-Ct value) using chicken intestinal organoids after inoculation with two cloacal (strain 1 and 3) and two lesion (strains 15 and 18) *E. cecorum* strains. The organoids were harvested at 3 or 6 h post inoculation.

			45—Ct Value			
			3 h		6 h	
Strain	Inoculation Dose (10^X^ CFU/Well)	N=	Mean	95% CI ^1^	Mean	95% CI
**1**	3	3	5.31	0.00–16.83	10.30	9.47–11.12
**3**	3	3	2.39	0.00–12.67	2.327	0.00–12.34
**15**	3	3	4.72	0.00–14.90	9.09	7.08–11.10
**18**	3	3	15.43	11.33–19.53	22.83	20.19–25.47
**3**	5	3	19.59	17.56–21.63	21.59	20.01–23.17
**18**	5	3	18.26	17.07–19.45	25.21	20.47–29.95
**1**	6	3	17.76	12.25–23.27	22.89	21.74–24.04
**15**	6	3	16.48	12.91–20.04	20.42	17.03–23.81
**1**	7	6	18.59	14.80–22.38	20.96	17.24–24.68
**3**	7	6	20.96	18.55–23.36	20.02	17.12–22.93
**15**	7	5	19.05	16.08–22.02	22.31	19.85–24.77
**18**	7	6	24.41	21.50–27.31	28.98	27.59–30.38

^1^ Confidence interval.

**Table 3:** Bacterial concentrations (CFU/mL) in an invasion assay using chicken intestinal organoids after treatment with an antimicrobial mixture and Triton-X100. The organoids were inoculated with two cloacal (strain 1 and 3) and two lesions (strains 15 and 18) *E. cecorum* strains and harvested at 3 or 6 h post inoculation.

			CFU/mL				
			3 h		6 h		
Strain	Inoculation Dose (10^X^ CFU/Well)	N=	Mean	95% CI ^1^	Mean	95% CI	
**1**	3	3	0	0–0	0	0–0	
**3**	3	2 or 3 ^2^	0	0–0	0	0–0	
**15**	3	3	0	0–0	0	0–0	
**18**	3	3 or 2 ^2^	0	0–0	0	0–0	
**1**	4	3	0	0–0	0	0–0	
**3**	4	3	0	0–0	0	0–0	
**15**	4	3	0	0–0	17	0–88	
**18**	4	3	0	0–0	0	0–0	
**1**	5	3 or 6 ^2^	0	0–0	1913	0–4292	
**3**	5	3 or 6 ^2^	1744	0–3710	2976	0–6800	
**15**	5	3	0	0–0	0	0–0	
**18**	5	3	0	0–0	0	0–0	
**1**	7	9	0	0–0	76	0–164	
**3**	7	6	2487	0–5012	4292	2821–5762	
**15**	7	9	59,267	0–126,782	111,978	55,256–168,700	
**18**	7	9	0	0–0	243,333	139,985–346,681	

^1^ Confidence interval. ^2^ Number of samples examined at respectively 3 h or 6 h post inoculation.

## Data Availability

The original contributions presented in the study are included in the article, further inquiries can be directed to the corresponding authors.
